# Diagnostic Performance of Indocyanine Green-Guided Sentinel Lymph Node Biopsy in Breast Cancer: A Meta-Analysis

**DOI:** 10.1371/journal.pone.0155597

**Published:** 2016-06-09

**Authors:** Xiaohui Zhang, Yan Li, Yidong Zhou, Feng Mao, Yan Lin, Jinghong Guan, Qiang Sun

**Affiliations:** Department of Breast Surgery, Peking Union Medical College Hospital, Chinese Academy of Medical Science and Peking Union Medical College, Beijing, 100730, China; AntiCancer Inc., UNITED STATES

## Abstract

**Background:**

The diagnostic performance of indocyanine green (ICG) fluorescence-guided sentinel lymph node biopsy (SLNB) for the presence of metastases in breast cancer remains unclear.

**Objective:**

We performed a meta-analysis to investigate the diagnostic performance of ICG-guided SLNB.

**Methods:**

Eligible studies were identified from searches of the databases PubMed and EMBASE up to September 2015. Studies that reported the detection rate of ICG fluorescence-guided SLNB with full axillary lymph node dissection and histological or immunohistochemical examinations were included. A meta-analysis was performed to generate pooled detection rate, sensitivity, specificity, false negative rate, diagnostic odds ratio (DOR) and a summary receiver operator characteristic curve (SROC).

**Results:**

Nineteen published studies were included to generate a pooled detection rate, comprising 2594 patients. The pooled detection rate was 0.98 (95% confidence interval [CI], 0.96–0.99). Six studies finally met the criteria for meta-analysis, which yielded a pooled sensitivity of 0.92 (95% CI, 0.85–0.96), specificity 1 (95% CI, 0.97–1), and DOR 311.47 (95% CI, 84.11–1153.39). The area under the SROC was 0.9758. No publication bias was found.

**Conclusion:**

ICG fluorescence-guided SLNB is viable for detection of lymph node metastases in breast cancer. Large-scale randomized multi-center trials are necessary to confirm our results.

## Introduction

In developed countries, breast cancer is a leading cause of cancer-related deaths in women aged 40 years and younger[1]. Early detection of breast cancer has been associated with reduced morbidity and mortality, compared to late detection[2]. In the early phase of breast cancer, breast cancer cells are mainly spread through the lymphatic system. Axillary lymph node dissection (ALND) has been used to evaluate lymph node status and identify the presence of metastases. However, ALND appears correlated with increased morbidity of lymphedema, pain, stiffness and shoulder weakness, seroma formation, vascular and brachial plexus injuries, and other complications. In patients with low-risk breast carcinoma, sentinel lymph node biopsy (SLNB) reportedly avoids ALND and thus reduces the complications associated with ALND[3].

Sentinel lymph nodes (SLNs) are the first lymph nodes that receive lymphatic drainage from the primary tumor [4]. SLNB is considered the standard care for patients without clinical or radiological evidence of axillary lymph node metastases in early-stage breast cancer[5]. A periareolar or interstitial injection of isotope and blue dye into the breast tumor is traditional in SLNB, and the 2 materials together are better than either alone [6].

A recent meta-analysis compared the traditional isotope-and-blue dye combination with 3 methods that are relatively new: indocyanine green fluorescence (ICG), contrast-enhanced ultrasound using microbubbles, and superparamagnetic iron oxide nanoparticles [4]. The newer methods showed clinical potential, but the false-negative rate of the compared methods ranged from zero for ICG to 30% for blue dye [4]. There was no ALND confirmation in some of the included studies, which potentially increased bias. Additionally, a jumping metastasis, or an abnormality in the lymphatic drainage pathway, makes a false negative inevitable, and an extrapolation of comparisons among different techniques is more difficult. In our opinion, the gold standard to identify whether a technique is reliable should be followed, that is, ALND plus histological or immunohistochemical confirmation.

In this study, we performed a meta-analysis to investigate the diagnostic performance of indocyanine green (ICG) fluorescence-guided SLNB in the presence of metastases in breast cancer, with ALND plus histological or immunohistochemical results as the reference.

## Methods

A systematic literature search was performed of the PubMed and EMBASE databases, for all relevant studies published until 24 September 2015. The study search was limited to patients and published in English. Keywords included “indocyanine green” and “breast cancer” and “sentinel lymph node”.

The selected studies conformed to 5 inclusion criteria: regional lymphadenectomy and pathological examination including hematoxylin-eosin staining or immunohistochemistry as the referenced standard; ICG fluorescence-guided SLNB and pathological examination as the diagnostic method; the sentinel lymph node (SLN) was the study’s major focus; pathological data of the reference standard and diagnostic method were both available; and included a defined subgroup of patients who underwent complete ALND after SLN dissection, regardless of the results of SLNB.

Studies were excluded for the following: reviews, meta-analyses and abstracts; animal studies; overlapping articles; lack of SLN identification rate of both SNL and patients’ statistical analysis; lack of pathological examination or unavailable pathological data; ICG combined with human serum albumin or other tracers (blue dye, radioisotope) while ICG data not separately reported; or the introduction of new technology.

### Data extraction and quality evaluation

The following data were extracted from the selected studies: first author; year of publication; country of origin; sample size; participants’ characteristics, tracers, concentrations, injected volumes, injected location, tumor characteristics; ICG-related adverse reactions, average number of detected SLNs, number of patients with successful fluorescence imaging, measures of test performance of ICG fluorescence-guided SLNB including true-positive, true-negative, and false-negative results. Six researchers were involved in data extraction.

The quality of each study was quantified using the quality assessment tool for diagnostic accuracy in systemic reviews (QUADAS, score from 0 to 14)[7]. QUADAS is a tool for assessing the evidence-based quality of studies for diagnostic accuracy.

### Statistical analysis

The measures of interest for effect included detection rates, sensitivities, specificities, diagnostic odds ratios (DORs), the summary receiver operator characteristic (SROC) curve, the area under the curve (AUC), and the Q* index. The closer the AUC is to 1.0, the better the diagnostic method. The Q* index is a statistical value defined by the point on the SROC curve where sensitivity and specificity are equal.

The statistical heterogeneity among studies was evaluated using Cochran’s Q statistic, *P*-values, and I^2^ statistics. Heterogeneity was considered significant at I^2^> 50%, or *P*< 0.05 (R GUI 3.0.1, R Project for Statistical Computing, USA). The sensitivity and specificity of each study was calculated by 2x2 contingency tables for correct diagnosis in the presence of lymph node metastasis.

The Mantel-Haenszel random-effects model was used to obtain the summarized detection rate, DOR, sensitivity, and specificity, considering the differences in patient characteristics, technical details, and operators’ experiences. The Q* index was subsequently produced from the SROC curve of all the included studies.

The publication bias for detection rate and diagnostic performance were explored by Harb or egger analysis, respectively, if sufficient studies were available[8]. *P*< 0.05 was considered statistically significant.

## Results

**Fig 1:**
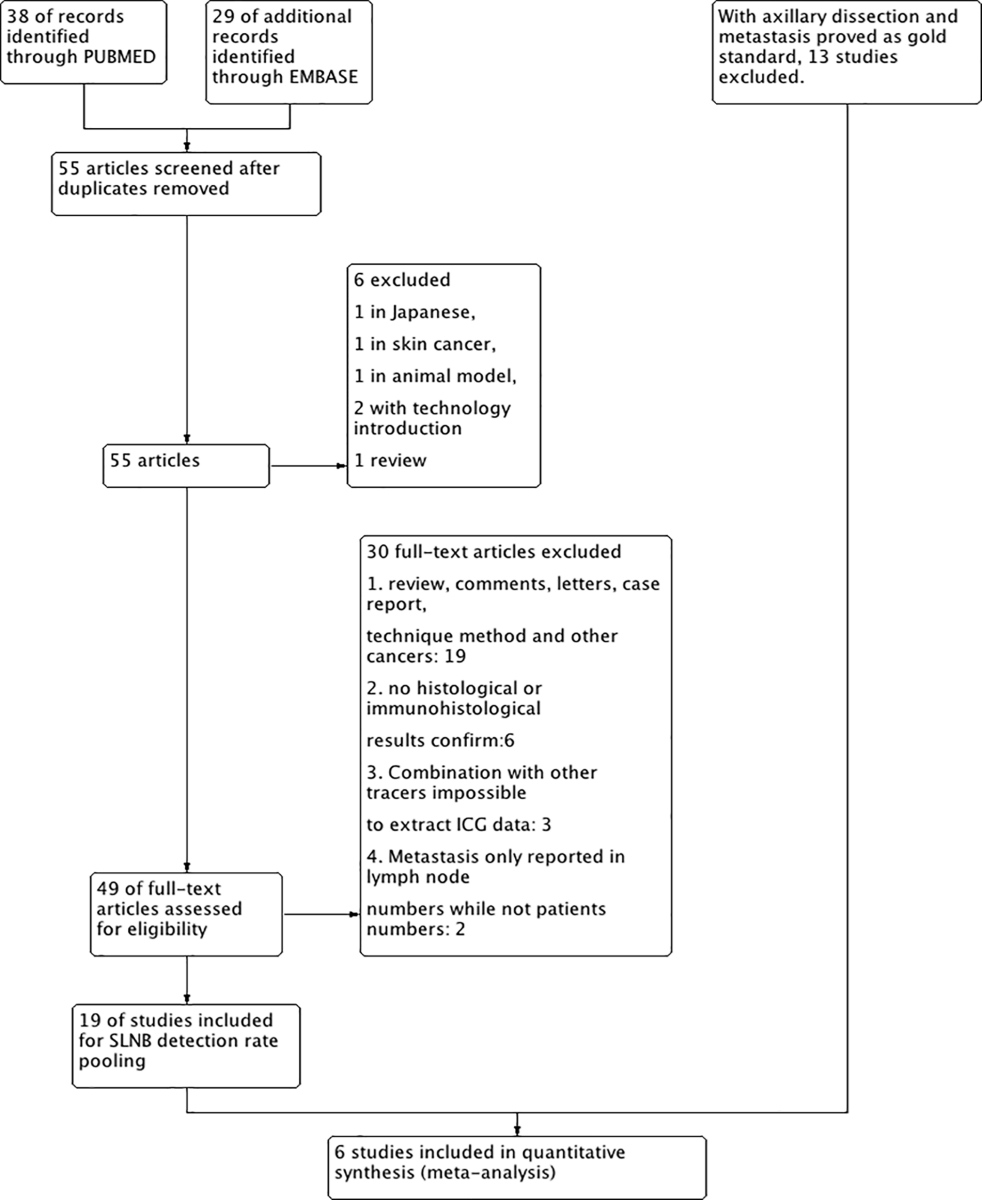
Flow chart for the selection of the included studies.

We identified 38 studies from PubMed and 29 studies from EMBASE. After removal of overlapping studies, languages other than English, animal studies, technology introductions, and reviews, 55 studies remained for full review (Fig 1). Of these, 36 full-text articles were additionally excluded: 25 abstracts, letters, case reports, or other kinds of cancers; 6 studies without histological or immunohistological results; 3 studies in which there was no separate data regarding ICG alone (i.e., not in combination with other tracers); and 2 studies reported only the numbers of metastasized lymph nodes but not the number of patients.

**Fig 2:**
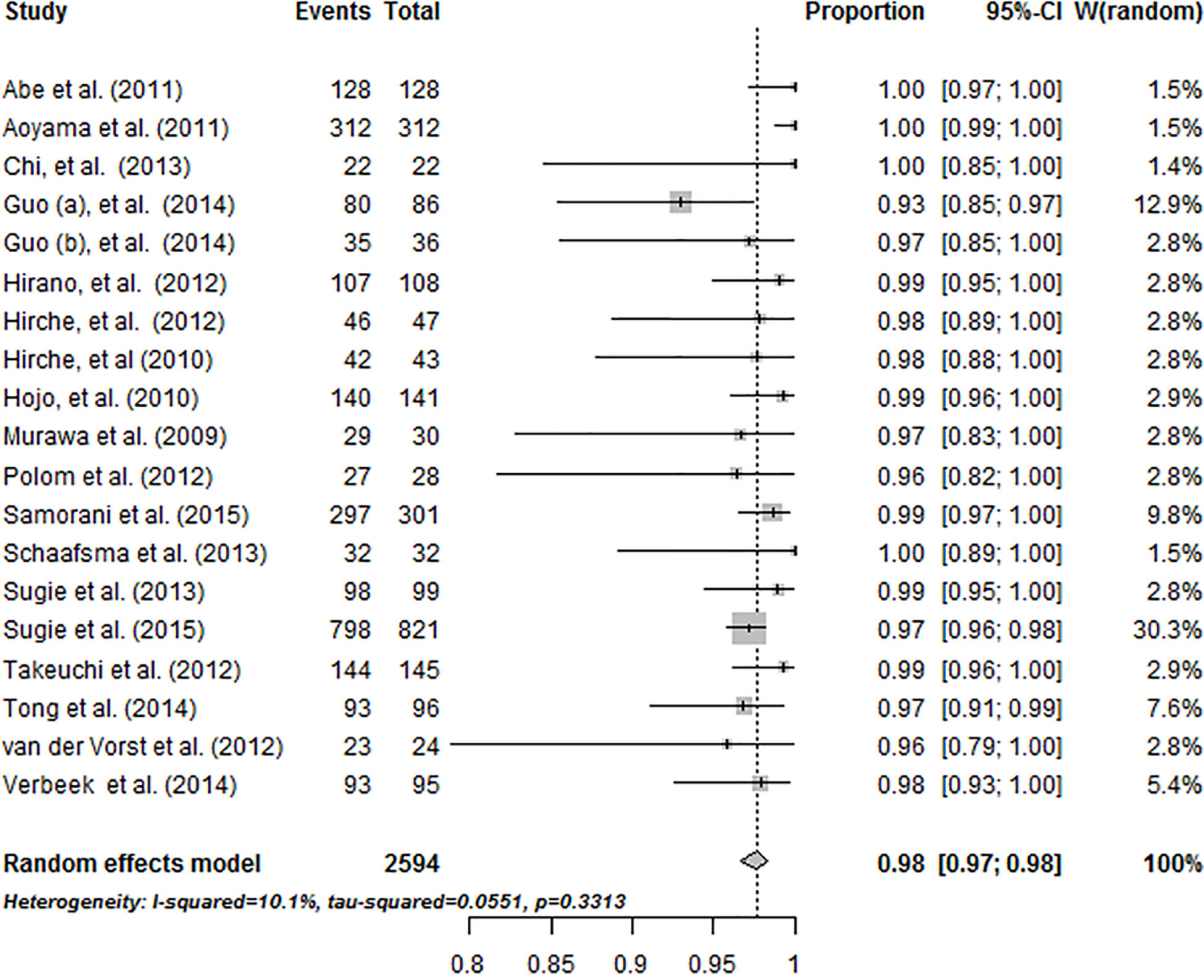
The pooled detection rates was 0.98 (95% confidence interval [CI], 0.96–0.99).

Finally, we included 19 qualified studies for pooling the detection rate (Tables 1 and 2) [9–27]. The 19 studies were published from 2009 to 2015, and included a total of 2594 patients. The pooled detection rate was 0.98 (95% confidence interval [CI], 0.96–0.99). The heterogeneity of the pooled detection rate was low: I^2^ value was 10.1%, *P* = 0.33 (Fig 2). Among 19 studies, there were only 6 studies [11–13, 15, 16, 18]in which after SLNB ALND was performed plus histology or immunohistochemistry as the reference, as required for our meta-analysis.

**Table 1:** Nineteen studies for the pooled detection rate analysis.

First author	Year	Country	Pnt/Cnt, n	Tumor stage	Other RI tracers used	Injection location	Dose (mL; mg/mL)
Abe	2011	Japan	128/1	T1-2, cN0	ICG+BD	Intradermal areola	0.15; 5
Aoyama	2011	Japan	312/1	cN0	ICG only	Subareolar & periareolar	5; 0.125
Chi	2013	China	22/1	T1-2N0M0	ICG only	Areola	0.5–2; 5
Guo [12]	2014	China	86/1	Tis-T2	ICG+BD	Subareolar	1; (1/20)
Guo [13]	2014	China	36/1	Tis-T2	ICG only	Subareolar	1; (1/20)
Hirano	2012	Japan	108/1	Tis-T4, cN0	ICG+BD cf. BD(along)	Subareolar	2.5; 2
Hirche	2012	Germany	47/1	N0-N3	ICG only	Subareolar	2.2; 5
Hirche	2010	Germany	43/1	N0-N3	ICG only	Subareolar	2.2; 5
Hojo	2010	Japan	141/1	Tis-T2, cN0	ICG+BD cf. ICG+RI	Tumor & sub-areolar	2; NA
Murawa	2009	Germany	30/1	T1-4	ICG for all, part with RC	Periareolar	1, 2, 3; 5
Polom	2012	Poland	28/1	N0-N1	ICG+RC cf. ICG+RC+HSA	Tumor or periareolar	1; 10
Samorani	2015	Italy	301/1	NA	ICG+radioactive	Periareolar	0.4–1.2; 5
Schaafsma	2013	Netherlands	32/1	NA	ICG+BD+RC	Tumor or periareolar	0.25–0.5 mg; NA
Sugie	2013	Japan	99/6	Tis-TX	ICG cf. BD	Subareolar	0.5–1; 5
Sugie	2015	Japan	821/12	T1-2	ICG +RC	Subareolar	1; 5
Takeuchi	2012	Japan	145/1	Tis-T2, cN0	ICG+BD for each	Subcutaneously into the periareolar area	1; 5
Tong	2014	China	96/1	Tis-T4, cN0	ICG+BD cf. BD	Subareolar	2; 5
van der Vorst	2012	Netherlands	24/1	cN0	(ICG+RC)+BD cf. no BD	Intradermal & periareolar	1.4; 500 μmoL
Verbeek	2014	USA & Netherlands	95/2	T1-4	ICG+RC	Periareolar& peritumor	1.6; 0.39

BD, blue dye; Cnt, medical centers; HSA, human serum albumin; NA, not available or not reported; Pnt, patient; RI, radioisotope

**Table 2:** Nineteen studies of the pooled detection rate analysis.

			SLN detection			
First author	Year	Pnt SLN rate	SLN No	mSLN	ALND confirm	Adverse effect
Abe	2011	100% (128/128)	(1–6)	3.1		NA
Aoyama	2011	100% (312/312)	(1–12)	3.41		Skin pigmentation for a certain period of time
Chi	2013	100% (22/22)	(1–6)	2.7	Yes	No side effect
Guo [12]	2014	93% (80/86)	NA	2.4	Yes	No adverse effect
Guo [13]	2014	97.2% (35/36)	NA	3.6	Yes	Tatoo effect last for one week & disappear in two weeks
Hirano	2012	99.1% (107/108)	(0–5)	2.2		NA
Hirche	2012	97.9% (46/47)	(1–3)	2.0	Yes	NA
Hirche	2010	97.7% (42/43)	NA	2.0	Yes	NA
Hojo	2010	99.3% (140/141)	NA	3.8		NA
Murawa	2009	97% (29/30)	NA	1.75	Yes	NA
Polom	2012	96.4% (27/28)	(1–5)	2		NA
Samorani	2015	98.7% (297/301)	(1–5)	1.94		No allergic reaction, (2.5%) developed paraesthesia.(3.2%) developed seromas
Schaafsma	2013	100% (32/32)	NA	1.5		NA
Sugie	2013	99.0% (98/99)	NA	3.4		NA
Sugie	2015	97.2% (798/821)	NA	2.3		No serious allergic reactions related to ICG injections reported in 833 consecutive patients. Grade 1 to 2 nausea or vomiting & pain observed in 8 (1.0%) & 6 (0.7%) patients, respectively.
Takeuchi	2012	99.3% (144/145)	NA			NA
Tong	2014	96.9% (93/96)	NA			NA
van der Vorst	2012	95.8% (23/24)	NA	1.5		NA
Verbeek	2014	97.9% (93/95)	(1–5)	1.9		NA

Cnt, medical centers; mSLN, mean number of SLN; NA, not available or not reported; Pnt, patient

**Fig 3:**
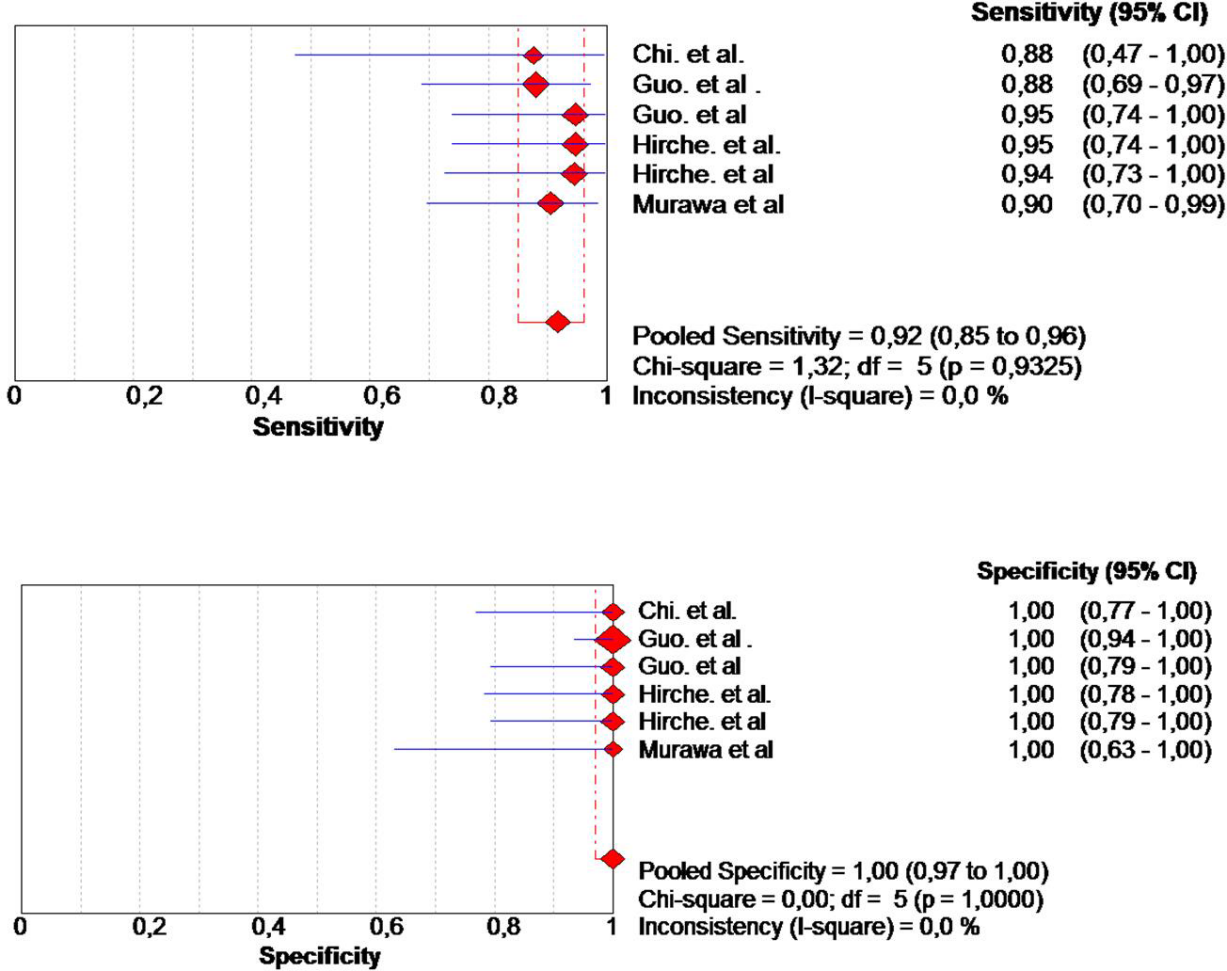
The pooled sensitivity was 0.92 (95% CI, 0.85–0.96), and the specificity was 1 (95% CI 0.97–1).

**Fig 4:**
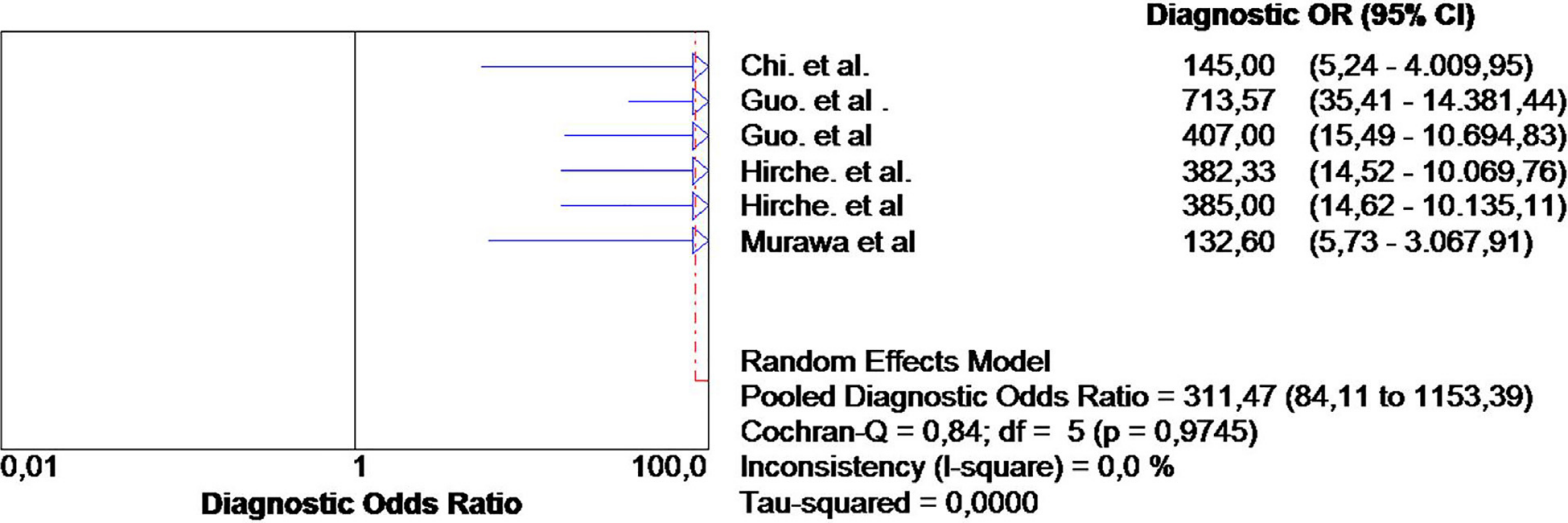
The pooled DOR was 311.47 (95% CI, 84.11–1153.39).

Altogether, 254 patients were included for the final analysis. The pooled sensitivity was 0.92 (95% CI, 0.85–0.96), and the specificity was 1 (95% CI 0.97–1). The heterogeneity of the pooled sensitivity analysis was low: I^2^ value was 0.0% (Fig 3), and the heterogeneity of the pooled specificity also had a I^2^ value of 0.0%. The pooled DOR was 311.47 (95% CI, 84.11–1153.39). The heterogeneity of the pooled studies was low: I = 0.0%, *P* = 0.97 (Fig 4).

**Fig 5:**
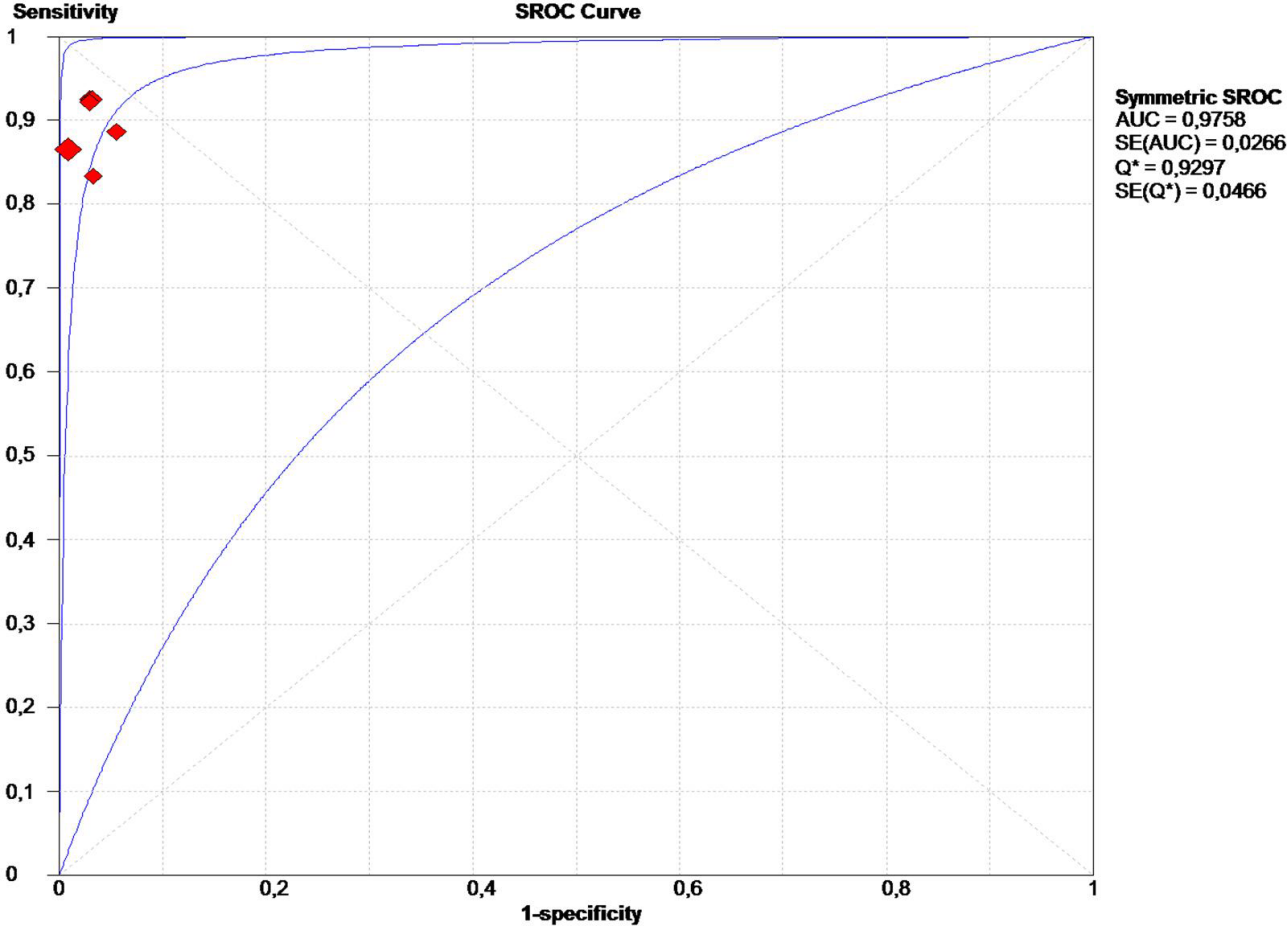
Summary of receiver operator characteristic curves.

**Fig 6:**
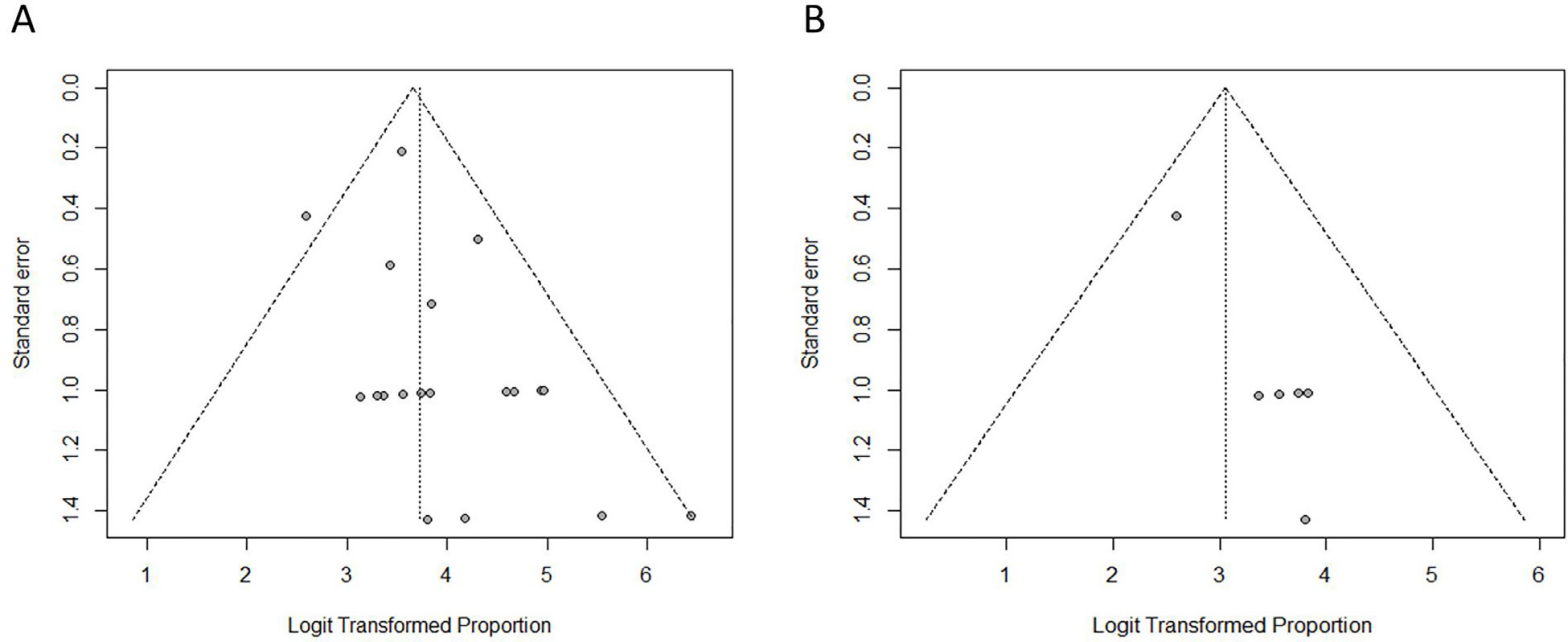
**Funnel plots:** (A) The 19 included studies for detection rate, (B) The 6 studies with ALND confirmation.

The AUC of the SROC was 0.9758 (Fig 5), and the Q* index = 0.93. Publication bias was not shown in any of the 19 studies analyzed early (Fig 6A), or the 6 studies in the meta-analysis (Fig 6B).

## Discussion

The 3 main findings of our study are, that ICG fluorescence-guided SLNB in breast cancer has a 98% detection rate for SLN; the pooled sensitivity and specificity were relatively high and the false-negative rate was relatively low; and in the presence of metastases, the diagnostic performance of this method is good, with relatively high sensitivity and very high specificity.

SLNB has been the first choice for axillary staging of patients with early breast cancer with clinically negative axillary lymph nodes. The good diagnostic performance of SLNB is essential for cancer staging, surgical treatment, and therefore substantially influences the prognosis. Currently, blue dye and radiocolloid are the two most common methods for SLNB. Blue dye is relative simple and inexpensive. However, the detection rate of blue dye is low[28]. Isotope has a relatively high detection rate, but its challenges include the handling and disposal of isotopes, high expense of storage, transport and even legislative issues which limit its wide use[29].

The use of ICG in SLNB has some advantages compared to isotope: lower cost, fewer adverse effects, and quick transcutaneous real-time visualization (within several minutes), facilitating the localization of the incision and the detection of SLNs during the surgery. In the clinical trial NSABP (National Surgical Adjuvant Breast and Bowel Project) trial B-32 that comprised 5611 patients, the combination of blue dye and radiocolloid showed a 97.1% detection rate for SLN, compared with 89.4% for radiocolloid alone and 70.2% for blue dye alone[30]. In another ALMANAC (Axillary Lymphatic Mapping Against Nodal Axillary Clearance) study including 842 clinically node-negative breast cancer patients, the combination of isotope and blue dye had a 96.1% detection rate, but that of either blue dye or isotope alone was 85.6%[6]. Our present study showed that ICG fluorescence alone may reach a detection rate of 98%, which is even better than combined isotope and blue dye. Similarly, a recent meta-analysis comparing ICG with blue dye showed that ICG was significantly better than blue dye with regard to SLN identification (odds ratio, 18·37)[4].

In our present study, the pooled sensitivity was 92%, and the false-negative rate was 8%. This is comparable to the results of the NSABP B-32, in which the false-negative rate for combined blue dye and radioisotopes was 9.8%. Another study that pooled data based on 8000 patients showed that the false-negative rates were 10.9% for blue dye alone, and 8.8% for radiocolloid alone[3]. The accuracy of the ICG method was not superior to the combination of blue dye and radiocolloid. However, when ICG was combined with another technique, the accuracy seems to improve; a false-negative rate of 4% was observed when ICG was combined with blue dye[13]. This clearly showed another way to increase the sensitivity of ICG.

In the present study, high DOR and AUC suggested a good diagnostic performance for ICG fluorescence-guided SLNB. However, these results should be interpreted cautiously. For example, the pooled specificity of the present study was 100%, which is not surprising since it is impossible to have false-positive sentinel node results—if a sentinel node is pathologically involved, the axillary lymph node basin is also involved. The high specificity is clearly responsible for the high DOR, as the DOR shows the overall performance of a diagnostic test, considering both sensitivity and specificity together. The SROC and its derivatives (AUC and Q*) are also measures of overall accuracy.

There were no severe adverse effects reported in the trials included in this study. Only two trials [9, 12]reported pigmentation, which lasted for several weeks. This makes ICG fluorescence-guided SLNB all the more attractive, when compared with the complications observed in ALND.

One main shortcoming of ICG fluorescence-guided SLNB is that indocyanine does not specially label tumor cells. Tumor-specific fluorescent probes which can selectively glow tumors, which preferably be administered topically, could significantly improve cancer detection and even removal in the surgery [31]: indeed, selectively highlighting tumor cells either by gamma-glutamyl hydroxymethyl rhodamine green, an enzyme commonly found in cancer cells[32]; or genetically label tumors in situ with green fluorescent protein[33, 34]; Spray-painting tumors by using fluorescent tumor-specific antibodies[35]. All of these new methods could potentially strengthen ICG fluorescence-guided SLNB, which deserves more studies in the future.

However, this meta-analysis also has several limitations. First, we only included 6 studies for the meta-analysis, mainly due to the reference standard demanded by the study design. Secondly, the technical limitations of the pathophysiological method used as the reference might have influenced the results. Finally, a false-positive rate is not possible in ICG fluorescence-guided SLNB, and this decreased the statistical power of the study.

## Conclusion

ICG fluorescence-guided SLNB is viable for detecting SLN in the presence of lymph node metastases in clinical node-negative breast cancer, and may allow the avoidance of ALND and its relatively greater complications. This study’s results warrant large-scale randomized multi-center trials and long-term follow-up for confirmation.

## Supporting Information

S1 PRISMA Checklist PRISMA checklist. (DOC)

S1 File Included papers in the search of EMBASE. (PDF)

## References

[1] Freedman RA, Partridge AH. Management of breast cancer in very young women. The Breast. 2013; 22:S176–S9. 10.1016/j.breast.2013.07.034 24074783

[2] Oeffinger KC, Fontham ET, Etzioni R, Herzig A, Michaelson JS, Shih YC, et al Breast Cancer Screening for Women at Average Risk: 2015 Guideline Update From the American Cancer Society. Jama. 2015; 314:1599–614. 10.1001/jama.2015.12783 26501536 PMC4831582

[3] Kim T, Giuliano AE, Lyman GH. Lymphatic mapping and sentinel lymph node biopsy in early-stage breast carcinoma: a metaanalysis. Cancer. 2006; 106:4–16. 16329134 10.1002/cncr.21568

[4] Ahmed M, Purushotham AD, Douek M. Novel techniques for sentinel lymph node biopsy in breast cancer: a systematic review. The Lancet Oncology. 2014; 15:e351–62. 10.1016/S1470-2045(13)70590-4 24988938

[5] Basso SM, Chiara GB, Lumachi F. Sentinel Node Biopsy in Early Breast Cancer. Medicinal chemistry. 2015. 10.2174/1573406412666151116144213 26567617

[6] Goyal A, Newcombe RG, Chhabra A, Mansel RE, Group AT. Factors affecting failed localisation and false-negative rates of sentinel node biopsy in breast cancer—results of the ALMANAC validation phase. Breast cancer research and treatment. 2006; 99:203–8. 16541308 10.1007/s10549-006-9192-1

[7] Whiting P, Rutjes AW, Reitsma JB, Bossuyt PM, Kleijnen J. The development of QUADAS: a tool for the quality assessment of studies of diagnostic accuracy included in systematic reviews. BMC medical research methodology. 2003; 3:25 14606960 10.1186/1471-2288-3-25 PMC305345

[8] Mueller KF, Meerpohl JJ, Briel M, Antes G, von Elm E, Lang B, et al Detecting, quantifying and adjusting for publication bias in meta-analyses: protocol of a systematic review on methods. Systematic reviews. 2013; 2:60 10.1186/2046-4053-2-60 23885765 PMC3733739

[9] Abe H, Mori T, Umeda T, Tanaka M, Kawai Y, Shimizu T, et al Indocyanine green fluorescence imaging system for sentinel lymph node biopsies in early breast cancer patients. Surgery today. 2011; 41:197–202. 10.1007/s00595-009-4254-8 21264754

[10] Aoyama K, Kamio T, Ohchi T, Nishizawa M, Kameoka S. Sentinel lymph node biopsy for breast cancer patients using fluorescence navigation with indocyanine green. World journal of surgical oncology. 2011; 9:157 10.1186/1477-7819-9-157 22132943 PMC3269998

[11] Chi C, Ye J, Ding H, He D, Huang W, Zhang GJ, et al Use of indocyanine green for detecting the sentinel lymph node in breast cancer patients: from preclinical evaluation to clinical validation. PloS one. 2013; 8:e83927 10.1371/journal.pone.0083927 24358319 PMC3865279

[12] Guo W, Zhang L, Ji J, Gao W, Liu J, Tong M. Breast cancer sentinel lymph node mapping using near-infrared guided indocyanine green in comparison with blue dye. Tumor Biology. 2014; 35:3073–8. 10.1007/s13277-013-1399-2 24307620

[13] Guo W, Zhang L, Ji J, Gao W, Liu J, Tong M. Evaluation of the benefit of using blue dye in addition to indocyanine green fluorescence for sentinel lymph node biopsy in patients with breast cancer. World journal of surgical oncology. 2014; 12:1–5. 25239029 10.1186/1477-7819-12-290 PMC4182872

[14] Hirano A, Kamimura M, Ogura K, Kim N, Hattori A, Setoguchi Y, et al A comparison of indocyanine green fluorescence imaging plus blue dye and blue dye alone for sentinel node navigation surgery in breast cancer patients. Annals of surgical oncology. 2012; 19:4112–6. 10.1245/s10434-012-2478-0 22782671

[15] Hirche C, Mohr Z, Kneif S, Murawa D, Hunerbein M. High rate of solitary sentinel node metastases identification by fluorescence-guided lymphatic imaging in breast cancer. Journal of surgical oncology. 2012; 105:162–6. 10.1002/jso.22075 21882198

[16] Hirche C, Murawa D, Mohr Z, Kneif S, Hunerbein M. ICG fluorescence-guided sentinel node biopsy for axillary nodal staging in breast cancer. Breast cancer research and treatment. 2010; 121:373–8. 10.1007/s10549-010-0760-z 20140704

[17] Hojo T, Nagao T, Kikuyama M, Akashi S, Kinoshita T. Evaluation of sentinel node biopsy by combined fluorescent and dye method and lymph flow for breast cancer. Breast. 2010; 19:210–3. 10.1016/j.breast.2010.01.014 20153649

[18] Murawa D, Hirche C, Dresel S, Hunerbein M. Sentinel lymph node biopsy in breast cancer guided by indocyanine green fluorescence. The British journal of surgery. 2009; 96:1289–94. 10.1002/bjs.6721 19847873

[19] Polom K, Murawa D, Nowaczyk P, Rho Y, Murawa P. Breast cancer sentinel lymph node mapping using near infrared guided indocyanine green and indocyanine green–human serum albumin in comparison with gamma emitting radioactive colloid tracer. European Journal of Surgical Oncology (EJSO). 2012; 38:137–42. 22130469 10.1016/j.ejso.2011.11.004

[20] Samorani D, Fogacci T, Panzini I, Frisoni G, Accardi FG, Ricci M, et al The use of indocyanine green to detect sentinel nodes in breast cancer: a prospective study. European journal of surgical oncology: the journal of the European Society of Surgical Oncology and the British Association of Surgical Oncology. 2015; 41:64–70. 10.1016/j.ejso.2014.10.047 25468752

[21] Schaafsma BE, Verbeek FP, Rietbergen DD, van der Hiel B, van der Vorst JR, Liefers GJ, et al Clinical trial of combined radio- and fluorescence-guided sentinel lymph node biopsy in breast cancer. The British journal of surgery. 2013; 100:1037–44. 10.1002/bjs.9159 23696463 PMC3681835

[22] Sugie T, Sawada T, Tagaya N, Kinoshita T, Yamagami K, Suwa H, et al Comparison of the indocyanine green fluorescence and blue dye methods in detection of sentinel lymph nodes in early-stage breast cancer. Annals of surgical oncology. 2013; 20:2213–8. 10.1245/s10434-013-2890-0 23429938

[23] Sugie T, Kinoshita T, Masuda N, Sawada T, Yamauchi A, Kuroi K, et al Evaluation of the Clinical Utility of the ICG Fluorescence Method Compared with the Radioisotope Method for Sentinel Lymph Node Biopsy in Breast Cancer. Annals of surgical oncology. 2016; 23:44–50. 10.1245/s10434-015-4809-4 26275781

[24] Takeuchi M, Sugie T, Abdelazeem K, Kato H, Shinkura N, Takada M, et al Lymphatic mapping with fluorescence navigation using indocyanine green and axillary surgery in patients with primary breast cancer. The breast journal. 2012; 18:535–41. 10.1111/tbj.12004 23009222

[25] Tong M, Guo W, Gao W. Use of Fluorescence Imaging in Combination with Patent Blue Dye versus Patent Blue Dye Alone in Sentinel Lymph Node Biopsy in Breast Cancer. Journal of breast cancer. 2014; 17:250–5. 10.4048/jbc.2014.17.3.250 25320623 PMC4197355

[26] van der Vorst JR, Schaafsma BE, Verbeek FP, Hutteman M, Mieog JS, Lowik CW, et al Randomized comparison of near-infrared fluorescence imaging using indocyanine green and 99(m) technetium with or without patent blue for the sentinel lymph node procedure in breast cancer patients. Annals of surgical oncology. 2012; 19:4104–11. 10.1245/s10434-012-2466-4 22752379 PMC3465510

[27] Verbeek FP, Troyan SL, Mieog JS, Liefers GJ, Moffitt LA, Rosenberg M, et al Near-infrared fluorescence sentinel lymph node mapping in breast cancer: a multicenter experience. Breast cancer research and treatment. 2014; 143:333–42. 10.1007/s10549-013-2802-9 24337507 PMC3899688

[28] Rutgers EJ. Guidelines to assure quality in breast cancer surgery. European journal of surgical oncology: the journal of the European Society of Surgical Oncology and the British Association of Surgical Oncology. 2005; 31:568–76. 10.1016/j.ejso.2005.02.008 16023942

[29] Stratmann SL, McCarty TM, Kuhn JA. Radiation safety with breast sentinel node biopsy. American journal of surgery. 1999; 178:454–7. 10670851 10.1016/s0002-9610(99)00230-5

[30] Krag DN, Anderson SJ, Julian TB, Brown AM, Harlow SP, Ashikaga T, et al Technical outcomes of sentinel-lymph-node resection and conventional axillary-lymph-node dissection in patients with clinically node-negative breast cancer: results from the NSABP B-32 randomised phase III trial. The Lancet Oncology. 2007; 8:881–8. 17851130 10.1016/S1470-2045(07)70278-4

[31] Bouvet M, Hoffman RM. Glowing tumors make for better detection and resection. Science translational medicine. 2011; 3:110fs10 10.1126/scitranslmed.3003375 22116932

[32] van Dam GM, Themelis G, Crane LM, Harlaar NJ, Pleijhuis RG, Kelder W, et al Intraoperative tumor-specific fluorescence imaging in ovarian cancer by folate receptor-alpha targeting: first in-human results. Nature medicine. 2011; 17:1315–9. 10.1038/nm.2472 21926976

[33] Kishimoto H, Zhao M, Hayashi K, Urata Y, Tanaka N, Fujiwara T, et al In vivo internal tumor illumination by telomerase-dependent adenoviral GFP for precise surgical navigation. Proceedings of the National Academy of Sciences of the United States of America. 2009; 106:14514–7. 10.1073/pnas.0906388106 19706537 PMC2732810

[34] Kishimoto H, Aki R, Urata Y, Bouvet M, Momiyama M, Tanaka N, et al Tumor-selective, adenoviral-mediated GFP genetic labeling of human cancer in the live mouse reports future recurrence after resection. Cell cycle. 2011; 10:2737–41. 21785265 10.4161/cc.10.16.16756 PMC3219541

[35] McElroy M, Kaushal S, Luiken GA, Talamini MA, Moossa AR, Hoffman RM, et al Imaging of primary and metastatic pancreatic cancer using a fluorophore-conjugated anti-CA19-9 antibody for surgical navigation. World journal of surgery. 2008; 32:1057–66. 10.1007/s00268-007-9452-1 18264829 PMC4378829

