# Supplementary material for: Diagnostic Performance of Indocyanine Green-Guided Sentinel Lymph Node Biopsy in Breast Cancer: A Meta-Analysis
Source: PLoS One. 2016 Jun 9;11(6):e0155597. doi: 10.1371/journal.pone.0155597 (PMC4900647; doi:10.1371/journal.pone.0155597)
Supplement: S1 File — (PDF) [file pone.0155597.s002.pdf]

1
Use of indocyanine green and methylene blue method in detection of sentinel lymph node in breast cancer patients with surgical navigation system: A multicenter experience
Chi C., Wang J., Zhang G., Wang Y., Tian J.
Molecular Imaging and Biology 2015 17:1 SUPPL. 1
2
The use of indocyanine green in sentinel lymph node biopsy in patients with breast cancer
Kou D.-Q., Qiu J.-D., Wang Q.-S., Wang J.-D.
Medical Journal of Chinese People's Liberation Army 2015 40:5 (392-395)
3
Assessing the effects of neoadjuvant chemotherapy on lymphatic pathways to sentinel lymph nodes in cases of breast cancer: Usefulness of the indocyanine green-fluorescence method
Tsuyuki S., Yamaguchi A., Kawata Y., Kawaguchi K.
Breast 2015 24:3 (298-301)
4
The retrospective study of sentinel lymph node biopsy by an indocyanin green fluorescence imaging
Ishii W., Iiduka R., Fujii K., Shimomae M.
European Journal of Surgical Oncology 2014 40:11 (S62-)
5
Evaluation of the benefit of using blue dye in addition to indocyanine green fluorescence for sentinel lymph node biopsy in patients with breast cancer
Guo W., Zhang L., Ji J., Gao W., Liu J., Tong M.
World Journal of Surgical Oncology 2014 12:1 (1-5)Article Number 290
6
Sentinel lymph node biopsy in breast cancer patients with previous breast augmentation surgery
Nagao T., Hojo T., Kurihara H., Tsuda H., Tanaka-Akashi S., Kinoshita T.
Breast cancer (Tokyo, Japan) 2014 21:5 (624-628)
7
Breast cancer staging: Sentinel node biopsy using a fluorescence navigation versus standard technique
Rzepka J.K., Misiek M., Bocian A., Zalewski K., Haduch J., Gozdz S.
Journal of Clinical Oncology 2014 32:15 SUPPL. 1
8
A novel approach for sentinel lymph node identification using fluororescence imaging and computed tomography lymphography in early-stage breast cancer patients
Abe H., Yamazaki K., Tokuda A., Ogawa M., Kawasaki M., Kameyama M.
Journal of Clinical Oncology 2014 32:15 SUPPL. 1
9
A systematic review of novel techniques for the performance of sentinel lymph node biopsy in breast cancer
Ahmed M., Purushotham A.D., Douek M.
European Journal of Surgical Oncology 2014 40:5 (617-)
10
Comparison of sentinel lymph node biopsy guided by the multimodal method of indocyanine green fluorescence, radioisotope, and blue dye versus the radioisotope method in breast cancer: A randomized controlled trial
Jung S.-Y., Kim S.-K., Kim S.W., Kwon Y., Lee E.S., Kang H.-S., Ko K.L., Shin K.H., Lee K.S., Park I.H., Ro J., Jeong H.J., Joo J., Kang S.H., Lee S.
Annals of Surgical Oncology 2014 21:4 (1254-1259)
11
One-year postoperative morbidity associated with near-infrared-guided indocyanine green (ICG) or ICG in conjugation with human serum albumin (ICG:HSA) sentinel lymph node biopsy
Murawa D., Polom K., Murawa P.
Surgical Innovation 2014 21:3 (240-243)
12
Breast cancer sentinel lymph node mapping using near-infrared guided indocyanine green in comparison with blue dye
Guo W., Zhang L., Ji J., Gao W., Liu J., Tong M.
Tumor Biology 2014 35:4 (3073-3078)
13

Evaluation of the benefit of using blue dye in addition to indocyanine green fluorescence for sentinel lymph node biopsy in patients with breast cancer
Guo W., Zhang L., Ji J., Gao W., Liu J., Tong M.
World journal of surgical oncology 2014 12 (290-)
14
Use of fluorescence imaging in combination with patent blue dye versus patent blue dye alone in sentinel lymph node biopsy in breast cancer
Tong M., Guo W., Gao W.
Journal of Breast Cancer 2014 17:3 (250-255)
15
Indocyanine green fluorescence-guided sentinel node biopsy: A meta-analysis on detection rate and diagnostic performance
Xiong L., Gazyakan E., Yang W., Engel H., Hünerbein M., Kneser U., Hirche C.
European Journal of Surgical Oncology 2014 40:7 (843-849)
16
99m Tc-Nanocolloid and Indocyanine Green: A Synergistic Alliance for a Better Result in Breast SLN Detectio
Peloso A., Ferrari A., Della Valle A., Gallo V., D'Addiego A., De Marco F., Mimmo A., Presazzi A., Cavenaghi G., Di Salvo I., Maestri M., Sgarella A., Aprile C.
European Journal of Nuclear Medicine and Molecular Imaging 2013 40 SUPPL. 2 (S215-)
17
Combined use of indocyanine green fluorescence and methylene blue dye versus methylene blue dye alone for sentinel lymph node biopsy in breast cancer patients
Wang S., Guo J., Yang D., Cao Y., Tong F., Zhou B., Liu P., Liu H., Cheng L., Liu M., Yang H., Xie F., Wang S.
Journal of Clinical Oncology 2013 31:15 SUPPL. 1
18
Indication of sentinel lymph node biopsy with breast-conserving surgery for ductal carcinoma in situ considering lymphatic flow
Yamaguchi A., Tsuyuki S., Okamura M., Kawata Y., Kawaguchi K., Kawaguchi N., Kohno Y.
Journal of Clinical Oncology 2012 30:27 SUPPL. 1
19
Will neoadjuvant chemotherapy affect the lymphatic routes to the sentinel lymph node? Investigation using indocyanine green fluorescence method
Tsuyuki S., Kawata Y., Yamaguchi A., Kawaguchi K., Okamura M., Kawaguchi N., Kono Y.
Journal of Clinical Oncology 2012 30:27 SUPPL. 1
20
Postoperative morbidity associated with near infrared guided indocyanine green (ICG) or icg in conjugation with human serum albumin (ICG:HSA) sentinel lymph node biopsy-one year analysis
Polom K., Murawa D., Murawa P.
European Journal of Surgical Oncology 2012 38:9 (883-)
21
Transparent plastic device; a new tool for near infrared guided indocyanine green sentinel node biopsy in breast cancer
Polom K., Murawa D., Rho Y.S.
European Journal of Cancer 2012 48 SUPPL. 1 (S209-)
22
Breast cancer sentinel lymph node mapping using near infrared guided indocyanine green and indocyanine green-human serum albumin in comparison with gamma emitting radioactive colloid tracer
Polom K., Murawa D., Nowaczyk P., Rho Y.S., Murawa P.
European Journal of Surgical Oncology 2012 38:2 (137-142)
23
High rate of solitary sentinel node metastases identification by fluorescence-guided lymphatic imaging in breast cancer
Hirche C., Mohr Z., Kneif S., Murawa D., Hünerbein M.
Journal of Surgical Oncology 2012 105:2 (162-164)
24
Sentinel Lymph Node Biopsy for Breast Cancer Patients Using Fluorescence Navigation with Indocyanine Green

Aoyama K., Kamio T., Nishizawa M., Ohchi T., Kameoka S.
[Article in Press] World Journal of Surgical Oncology 2011 (157-)
25
Breast Cancer Sentinel Lymph Node Mapping Near Infrared Guided Indocyanine Green and Indocyanine Green Human Serum Albumin in Comparison with Gamma Emitting Radioactive Colloid Tracer
Rho Y.S., Siddiqui F., Murawa D., Polom K.
European Journal of Medical Research 2011 16 SUPPL. 1 (93-94)
26
Sentinel lymph node navigation surgery with indocyanine green fluorescence in early breast cancer
Kassim K., Sugie T., Takada M., Ueno T., Yamashiro H., Tsuji W., Takeuchi M., Toi M.
European Journal of Cancer 2011 47 SUPPL. 1 (S375-)
27
Sentinel lymph node biopsy in breast cancer patients with previous breast augmentation surgery
Nagao T., Hojo T., Kurihara H., Tsuda H., Tanaka-Akashi S., Kinoshita T.
[Article in Press] Breast Cancer 2011 (1-5)
28
Comparison of indocyanine green (ICG) fluorescence imaging plus blue dye and blue dye alone in sentinel node navigation surgery (SNNS) for breast cancer
Hirano A., Shimizu T., Kamimura M., Ogura K., Kim N., Setoguchi Y., Okubo F., Inoue H., Miyamoto R., Kinoshita J., Ogawa K.
Journal of Clinical Oncology2011 29:15 SUPPL. 1
29
Preliminary experiences of sentinel lymph node biopsy for early breast cancer by a new camera system simultaneously capturing color and near-infrared fluorescence
Sugimoto T., Sato T., Hokimoto N., Funakoshi T., Inoue M., Ogata H., Hanazaki K.
European Journal of Cancer, Supplement 2010 8:3 (153-)
